# The notion of free will and its ethical relevance for decision-making capacity

**DOI:** 10.1186/s12910-019-0371-0

**Published:** 2019-05-08

**Authors:** Tobias Zürcher, Bernice Elger, Manuel Trachsel

**Affiliations:** 1 Institute for Biomedical Ethics and History of Medicine (IBME), University of Zurich, Winterthurerstrasse 30, CH-8006 Zürich, Switzerland; 2 Center for Legal Medicine, University of Geneva, Geneva, Switzerland; 3 Institute for Biomedical Ethics, University of Basel, Basel, Switzerland; 4 Psychiatric Outpatient Services, Thun, Switzerland

**Keywords:** Informed consent, Decision-making capacity, Competence, Ethics, Free will, Autonomy, Authenticity, Compatibilism, Harry Frankfurt

## Abstract

**Background:**

Obtaining informed consent from patients is a moral and legal duty and, thus, a key legitimation for medical treatment. The pivotal prerequisite for valid informed consent is decision-making capacity of the patient. Related to the question of whether and when consent should be morally and legally valid, there has been a long-lasting philosophical debate about freedom of will and the connection of freedom and responsibility.

**Main text:**

The scholarly discussion on decision-making capacity and its clinical evaluation does not sufficiently take into account this fundamental debate. It is contended that the notion of free will must be reflected when evaluating decision-making capacity. Namely, it should be included as a part of the appreciation-criterion for decision-making capacity. The argumentation is mainly drawn on the compatibilism of Harry Frankfurt.

**Conclusions:**

A solution is proposed which at the same time takes the notion of free will seriously and enriches the traditional understanding of decision-making capacity, strengthening its justificatory force while remaining clinically applicable.

## Background

Informed consent is central to the legitimation of medical treatments. Obtaining the patient’s informed consent is not only a legal prerequisite. Beyond that, it is considered a moral duty because it reflects the healthcare professionals’ respect for personal autonomy and the individual’s right to self-determination [1–5]. “[In] the late1950s […] medicine became self-aware and self-critical about its paternalistic ways and began to take the rights of the patient seriously: patients, it was determined, should be truthfully informed about their diagnosis and the nature of available treatments (including harms and risks)” ([6] p. 23). However, why has respect for autonomy become such central moral principle? A common way to comprehend this, is that autonomy can be conceived as axiomatically valuable [7]. However, this cannot be the only reason, because autonomy has not been an important moral principle in other times and cultures including eras that were not dominated by medical paternalism. Another reason for the increased importance of respect for autonomy in medicine could be that respecting patients’ autonomy fosters favorable treatment outcomes. In addition to axiomatically setting the importance of autonomy, we can therefore bring in a second argument based on a very general duty to provide good therapy. This argument states in the first normative premise that (1) in medicine, good therapy is essential. The second empirical premise is that (2) respect for autonomy promotes good therapy - and vice versa: disregarding autonomy is detrimental to good therapy (ceteris paribus). From this, we can now conclude that respect for autonomy is an obligation/objective.

We consider the first premise, in this general formulation, to be indisputable. The second premise is more demanding. Although it has to be still empirically shown if and to what extent autonomy instantiated in informed consent fosters well-being, we have numerous indications for this assumption: Appropriate informed consent procedures are important for augmenting mutual trust, and so provide the basis for a good and stable alliance between patient and health care professional. For example, in the case of psychiatric treatments, it has been shown that a strong therapeutic relationship is among the most important factors for a successful treatment outcome (e.g., [8–10]. According to Beahrs and Gutheil ([11] p. 6),

[…] the duty to provide informed consent fosters shifts from indoctrination to information sharing and from paternalism to respect of patients’ autonomy. In so doing, we help to access, validate, empower, and challenge our patients’ own natural strengths so that they can use these strengths toward more effective self-help, the sine qua non of a positive treatment outcome.

Informed consent further requires that health care professionals explain diagnostic findings and provide information about the illness/disorder itself, as well as reasons for and characteristics of the proposed treatment, such as aims, benefits, expected course, and expected duration [11]. In addition, information about alternative therapy options, potential risks, side-effects, and consequences of refusal should be disclosed to the patient [12–14].

Just as respect for autonomy is expressed in the duty to get informed consent, so informed consent presupposes the patient’s decision-making capacity (DMC). Therefore, DMC is seen as the key element for informed consent in medicine [15]. On this clinical level, this does not cause any fundamental problems. However, problems arise on a more conceptual level and will be discussed in the following.

In the present contribution, it is our aim to introduce a philosophically crucial element for the justification of actions into the debate on autonomy, informed consent, and DMC: the notion of free will. We have argued above that respect for autonomy is crucial. Now we can build another argument on the conclusion of this argument (or on the establishment of an axiomatic value of autonomy): Here we state (1) that the duty to respect autonomy requires that this value be implemented in clinical instruments (such as DMC). We claim that (2) DMC considers aspects of autonomy that are necessary but not sufficient. With assumption (3) that free will completes the DMC criteria, we can conclude that free will is a prerequisite for a justified responsible decision in practice.

## Main text

Free will is largely considered as a necessary condition for moral responsibility. O’Connor and Franklin note (with many references to literature) that “the kind of control or sense of up-to-meness involved in free will is the kind of control or sense of up-to-meness relevant to moral responsibility” [16]. We will see later that it is controversial what conditions must be met in order to assume control which is sufficient for free will. In particular, it is controversial whether this should include the ability to choose otherwise, which would be made impossible by the truth of determinism. A minority of thinkers in the free will debate denies that control is sufficient for free will, but nevertheless assumes that this control is necessary to be morally responsible [17]. We will argue later that it makes sense to understand free will independently of the question of determinism. Therefore, we stand for the view that a (yet to be explained) form of control is sufficient for free will, which in turn is a necessary condition for responsibility.

Although they share common ground, the debates of DMC and free will have been usually taken place separately. This may be because of prejudices by one part of scholars judging the classical free will debate detached from the real world, or by the other part of scholars dismissing all pragmatic accounts to be theoretically unfounded. We are convinced that both views are wrong. We believe these lines of thoughts to be closely related and providing the potential for mutual understanding. The connection, envisaged in this paper, should, on the one hand, meet the theoretical profoundness of the philosophical debate on free will, and on the other hand, be applicable in clinical practice when it comes to the evaluation of DMC. Another misunderstanding that we must clear up is the following: It does not follow from the practical necessity of an instrument (such as DMC) that this instrument legitimizes itself. Nor do legal claims (as they are formulated in many ways in the case of respect for autonomy) legitimize themselves. Even for philosophers of law who advocate a conceptual separation of law and ethics, it is by no means impossible to question the ethical legitimation of law. Accordingly, for example Leslie Green, one of the most important contemporary legal philosophers (and legal positivists), states that “no legal philosopher can be only a legal positivist” [18]. What applies to legal norms applies a fortiori to instruments which are not themselves legal norms, to which soft law belongs or which standardize professional duties. With this assumption we oppose a doctrine that can be described as ethical nihilism in the philosophy of law. With regard to DMC as an instrument of clinical practice, we are convinced that it is only justified morally if freedom of will is not excluded. In the first part of the present contribution, we present an overview of the classical understanding of DMC as well as of the philosophical approaches to free will. In the second part, we further examine positions on free will, argue for compatibilism introducing the theory of Harry Frankfurt and defend it against recently raised criticism. In the third part, we take a closer look at various types of inner and outer constraints and their implication for the assessment of patient’s appreciation as one important criterion for DMC. In the conclusion section, we integrate our findings.

### Classical criteria for decision-making capacity

The following criteria for DMC are widely accepted [19]: (a) the *ability to understand* the relevant information. *Understanding* consists of the ability to comprehend treatment-related information concerning the present disorder, treatment options, and the related risks and benefits [19]. (b) the *ability to appreciate* the disorder and the medical consequences of the situation. *Appreciation* refers to the ability to acknowledge the nature of the disorder and the possibility that treatment may be beneficial. This differs from the understanding standard in that it requires the patient to apply the information abstractly to his or her own situation [19]. It takes into account that one and the same diagnosis or therapy differ in the way they affect two persons in their different living conditions and attitudes towards life. (c) the *ability to reason* about treatment choices. *Reasoning* requires a certain consistency of beliefs and the ability to derive conclusions from premises (although there are conceptions which go much further by requiring true beliefs as premises; see [3]. (d) the *ability to communicate* a choice.

Many mental and physical disorders can impact significantly on DMC [20–23]. “Decision-making incapacity is associated with a broad range of clinical conditions that include various forms of dementia, delirium, organic amnestic syndromes, brain injury, and disorders of consciousness such as coma, vegetative and minimally conscious states, as well as psychiatric diseases such as schizophrenia or severe depression, or medically- or illness-induced impaired consciousness in critically unstable patients who are too ill to participate in decision-making” ([5], p. 360).

DMC can change with the fluctuation of symptoms, as well as over time and across different situations [5, 20]. For example, a person may be deemed to have DMC for matters of everyday life (e.g., what to eat) but may not be sufficiently capable of making decisions regarding medical treatment (referred to as *decisional relativity* by Buchanan and Brock [24]. Because DMC is thus decisional relative and furthermore not time-invariant, its validity is related to the date of the evaluation [13]. Not the criteria for DMC are variable, but the difficulty of patients to meet them dependent on their clinical condition (in this regard, we shall see later an accordance with free will). For instance, both, the determination on which finger a drop of blood has to be taken, and the decision on a chemotherapy, must be sufficiently understood, appreciated, reasoned and communicated. Neglecting the simple cases is not due to ignorance with regard to the justifying conditions but to the comparatively harmless consequences.

Free will: why we need a compatibilist understanding

The fundamental importance of free will for the legitimation of any treatment decision by a patient is widely acknowledged (e.g., [25]). To get a basic understanding of what freedom of will is, consider the following cases: First, think of a prisoner who is almost entirely unable to do what she wants. Nevertheless, in her imagination she contemplates different scenarios, and, by virtue of this, has many desires. Now, compare the prisoner with a billionaire, who, with huge financial resources, could put a large part of his wishes into effect. However, the billionaire is suffering from an episode of acute mania that interferes with his ability to resist any of his impulses to buy sailing yachts. While the prisoner lacks *freedom of action* – while her will is not necessarily unfree because of the fact of being incarcerated – the billionaire lacks free will. A person can enjoy freedom of action yet still not wanting something freely (and vice versa). Both persons in these cases lack full autonomy (either because of an outer constraint, as the prison, or of an inner constraint, as the mania).

What exactly is *freedom of the will*? Why is it that a mania destroys it? The ability to form a will freely, i.e. to be the author or owner of one’s will means to experience a certain degree of control. Indeed, freedom of will (in the Western debate) has always been related to the concept of control [16]. In the free will debate, there are two principal opposing positions, which differ in terms of the consequences the notion of determinism would have for freedom (see e.g., [26–28]). *Determinism* is the thesis that certain conditions entail the occurrence of every present and future event (as in *fatum*, god’s foreseeing, or most importantly, the laws of physics plus events in the past). We do not consider here any ideas which presuppose the existence of a personal destiny or God, and therefore limit ourselves to a determinism based on natural law [29]. *Compatibilism* holds that we can be free despite or, according to some versions, precisely because of the truth of determinism [31, 32], for an overview see [30]. Conversely, *incompatibilism* denies that determinism and free will can both be true. Among incompatibilists, the *libertarians* argue that free will is possible and determinism is false while the *hard incompatibilists* (or *hard determinists*) assert that free will is impossible because determinism is true [33].

We contend that a compatibilist framework is most convincing. The compatibilist theory which we defend does not make any metaphysical assumptions, i.e. it is irrelevant as to whether determinism is true or false. This refers to many variations of compatibilism, but – by definition – of no incompatibilist theory. Accordingly, the theories which are neutral with regard to the truth of determinism are more probably right and applicable because also a libertarian must – in addition to showing the truth of indeterminism – argue under what circumstances a person can be reasonably connected to her (allegedly) own will (see also [23]).

One could ask oneself whether the problems that arise around the truth or untruth of determinism are not of a purely theoretical nature. Could it be that this dispute represents an exclusively metaphysical problem that is meaningless to medical ethics? We think that there is a big misunderstanding because it either reveals a false understanding of metaphysics (and thus a false understanding of what philosophy does) or a false understanding of the difference between the premises of hard incompatibilism and compatibilism. Freedom of the will is by no means a “purely” metaphysical notion. Strictly speaking, the concept of free will implies metaphysical questions, but it is not limited to them. It is a highly practical term. It is not at all unusual that key concepts of our practical life are influenced by metaphysical assumptions. Consider for example the concepts of “knowledge” and “justice”: Both are terms with high relevance to our practical life and both are influenced by metaphysical assumptions. For example, our thoughts on these terms may be influenced by whether we assume that the world is intelligible at all (whether there are fundamental sensory data or pure reason etc.) or whether or not there is absolute justice (owing to the existence of God or moral realism etc.). No matter how we face up to these assumptions, we position ourselves.

On this background, we have a burden-of-proof argument for compatibilism: It is an undecided problem whether determinism is true or false [34]. Balaguer notes that neither the “Copenhagen Model” nor Bell’s theorem, nor a multiverse theory, can clearly prove that indeterminism is right or wrong. Therefore, it is the case that there are “no good arguments for or against determinism” [34]. Moreover, it remains controversial whether indeterminism at a quantum physical level holds in a way that could relevantly affect a person’s deliberations and decisions [35]. However, even if we would follow an interpretation of quantum physics according to which indeterminism is true there would remain two problems: First, it would be to clarify if and how quantum indeterminism affects neurological and (or) our deliberational process. Second, and more important, the task of assigning the will to a person must (nevertheless) be spelled out. A certain act of will must belong to the very person who expresses it, in such a way that the will is related to that person. This is a common task for both, libertarianism and compatibilism [16]. This requirement, which has to be assessed in every individual case, can be expressed namely as non-randomness of one’s will [34]. So, pragmatically, and unaffected by the problem of determinism, we should concentrate on the hardly uncontested part of the free will debate, which consists – as we have stated above – in the widely shared assumption that a certain kind of control over one’s actions is necessary for free will. Indeed, physicians who are inclined to be indifferent regarding determinism [36, 37] have grasped that they should focus on patients control over their will. Here, we present the arguments that support clinical practice, and we argue that hard incompatibilism and libertarianism may play a significant role in the general free will debate, but that they are of negligible significance for the discussion of DMC and thereby informed consent.

### Frankfurt’s theory of free will

We contend that Harry Frankfurt’s famous compatibilist theory is both, theoretically well founded and applicable in practice. Frankfurt proposed a theory that simply assumes us to be free when “what we want is what we want to want” [38]; for the latest discussion of Frankfurt’s conception see [30]. Frankfurt draws distinctions between different types of desires. *First-order-desires* are those related to actions (e.g., a desire to wash my hands); s*econd-order-desires* are desires related to desires (e.g., a desire to have the desire to wash my hands). Free will consists of what Frankfurt described as a “certain volitional unanimity” ([38] p. 15). If a person succeeds in making a second-order-desire effective, then he or she enjoys free will. In this case, an action motivating first-order desire is exactly the desire by which we want our action to be motivated [38], in which case there is no substantial “inner” conflict of any sort.

The above-mentioned examples clearly show the similarities between the classical DMC criteria and Frankfurt’s understanding of free will: Persons enjoy free will when they are able to evaluate their desires by withholding some of them and identifying one that seems the best choice “overall”. Accordingly, with respect to DMC, these persons understand the relevant information and can appreciate what is at stake. Frankfurt coined the term “wholeheartedness” to describe the state in which a person comprehends his or her motives to be in harmony with each other and where no motivating desires are imposed against that person’s will ([39] p. 165). Reaching this harmony doesn’t mean that individuals – if they want freely – eliminate all their weaker second-order-desires. The required reflection capability only ensures that they pick out the crucial desire in the light of their own picture of themselves or their determining aims in life. Frankfurt claims that practical reasoning is conceptually connected to goals and to what we fundamentally care for [38]. In his more recent writings, Frankfurt emphasizes more strongly that desires relevant to freedom must express what the person is at heart [38]. In this regard, his position qualifies as a “self-expression theory” which must be differentiated from “control-based theories” (for a good overview see [40]). For a self-expression theory, it is essential that it assumes the existence of a set of properties that are constitutive for the person’s deep self. As Sripada notes, the self is the “cluster of attitudes that specify what matters most to her and what is for her most worth pursuing” ([40] p. 785). Frankfurt defines them as the source of our practical reasons [38]. What we care about defines what goals we have and this differs from what we ordinarily desire in so far as goals reflect these desires with which we identify. Persons do not simply have desires but are able to make up their minds to reflect on desires, to identify with a specific desire, and to withhold assertion to others. [38].

It is conceivable that a person has two or even more rivaling second-order-desires. That is simply what it means to decide in uncertainty or dealing with ambivalence. What is important is that considerations of these desires come to an end after a while, by identifying the strongest of these desires (in the next section we will discuss a related objection).

Frankfurt’s theory has not stayed undisputed. In the following section we will discuss and rebut four objections that we consider particularly relevant for the focus of our argumentation: the first relates to the relevance of the causal explanation of desires, the second objection concerns the question of whether reflection can come to an end in time, the third objection concerns whether the requirements for reflection meet our usual understanding of well-considered and (therefore) free decisions, and the fourth objection criticizes Frankfurt’s approach on the grounds that it is “impractical”.

### Four objections to the Frankfurtian approach and their rebuttal

Are we free, when we – after reflection – accept a desire that is actually *caused* by a second-order desire? Wouldn’t this be then a sort of illusion of rationality or pseudo-reflection? Consider the following example: I want to eat an ice cream cone and I think of myself as a person who should be allowed to eat one ice cream cone once a week, and therefore fully endorse this actual (first-order) desire. Let us assume that I didn’t eat any ice cream for 1 week and I’m going to have one cone right now. But, from a third-person perspective, we would know that this reflective endorsement actually resulted from bodily processes (urge for sugar, hunger, positive emotions when eating ice cream during childhood among others) and is therefore nothing more than a kind of by-product. We can counter this as follows: Remember that a second-order desire is defined as a desire about a desire and not about an action. As such, it is necessarily a reflective process as it involves a certain self-distance to what is going on in one’s own mind. From a compatibilist point of view, the causation of a desire – by experience, by biological urge, or any other way – does not destroy freedom. We would only come to this conclusion if we hold an incompatibilist standpoint. But as Frankfurt states: “Insofar as we are governed by causal forces, we are not omnipotent. That has no bearing, however, upon whether we can be free” ([38] p. 178). Presumably, it is just as much a (disguised) paternalistic intuition as a fear from determinism, what may lead to judge a person as unfree, when the causal history of a desire seems to be (too) transparent. In fact, we may be inclined to deny someone’s freedom, when the almost obvious “mechanical apparatus” is visible, all the more if it involves any self-damaging actions, as in the case of drug use. In his famous example of the willing addict, Frankfurt illustrates a man, who willfully (by second-order reflection) wants to have the desire (first-order) to take drugs although it would not be possible at all to have the desire to give up the drug because of the physical dependence [38]. In other words, although the renunciation of drugs is not really possible for X, i.e. he does not control the desire, he has nevertheless fully embraced it. In contrast to the unwilling addict, he is therefore responsible for his desire to take the drug and his corresponding actions (for a strong defense of self-express theories against control-based theories see [40].

The willing addict is not that unrealistic as for instance, socially well integrated cocaine addicts could have a harmonious wish to take the drug if the first-order wish to consume cocaine is compatible with their overall life-plan. In the case of many other drug addicts who live in desolate social conditions, this might be rare. The here presented compatibilist conception of free will is strictly “internal” to an agent’s own desires, which means it does not presuppose certain (objective) values or does not require to assess a patient’s life-plans (for the discussion of objective values in Frankfurt’s account, see [42]. This means, most importantly, that patients are not necessarily unfree if – as part of their wish forming – they take into account an addiction or a disease. It would be unjustified paternalistic to deny DMC in such cases by default. Unfreedom would exist if patients were under the acute influence of pain or fear “forcing” them to refuse treatment or rehabilitation, even though – as a second-level desire – they would like to accept professional support.

Especially in the case of obvious manipulations we seem to deny the freedom of the manipulated person. If, like in the following thought experiment, our wishes were caused by a malicious neurosurgeon, they do not really seem to be our very own [43]. The question is whether we can be manipulated and still be free and responsible. In the case mentioned above, responsibility seems suspicious to us because we suspect that there is another self, the true self, which is now being deceived by the malicious neurosurgeon. If this is indeed the case, then the person is not free according to Frankfurt’s theory, because the desire created by the malicious neurosurgeon is not an expression of the real self. However, if there is no such betrayed self left, then the person is free. This may be an astonishing, perhaps even frightening conclusion. But let us remember what it would mean to be responsible for who we are. We should be able to rise above all determinants and create ourselves. However, our education, to name just one example, is a causal destiny of our self that we ourselves cannot really influence, let alone control. Frankfurt’s theory does not make freedom and responsibility dependent on whether we are responsible for who we are. If any form of determinism would be true, we wouldn’t be anybody. Frankfurt states: “We are not fictitious characters, who have sovereign authors; nor are we gods, who can be authors of more than fiction. Therefore, we cannot be authors of ourselves... We can be only what nature and life make us, and that is not so readily up to us” ([44] p. 10).

The second objection we would like to discuss is commonly referred to as the regress problem. It states that—given Frankfurt’s hierarchical approach of the will—we never get out of the loop of reflection (in a third or even higher order) and therefore cannot determine a certain desire to be effective. Frankfurt objected that the first-order desire could be approved by the second (reflective) stage “without reservation” [39]. By identifying fully (wholehearted) with one’s own will, a higher-level, non-concluding reflection would become pointless. In addition to Frankfurt’s defense based on conceptual considerations, however, we should above all consider the phenomenology of such an impending over-reflective will. How would we assess a person who never stops reflecting on his or her own will? Insofar as this occurs at all, the case does not pose a particular problem because the person would obviously be incapable of making a decision. It should be noted that such decisions may be rare and that losing oneself in circles of reflection could become a case of (pathological) inability to determine one’s own will. However, the reflection of desires doesn’t entail perfect rationality, comprehensive knowledge of circumstances, or even infallibility. What is required is a certain overview of a person’s desires in the light of his or her fundamental values and aims. And this is precisely what a physician should consider when evaluating DMC and what we will further explore in the next section.

According to the third objection, Frankfurt states in his theory that many of the almost universally considered autonomous actions would turn out to be non-autonomous if agents have never reflected upon their desires [7]. This argument is based on the assumption that the scope of the term autonomy should not be *revisionary* (as would be assumed by incompatibilists), but should rather remain *descriptive*, which means that it is based on the (presumptive) commonly shared concept of the possibility of autonomy (for the methodological background of descriptive and revisionary approaches, see [45]. Beauchamp claims that “ordinary choices qualify as autonomous even when persons have not reflected on their preferences at a higher level and even when they are hesitant to identify with one type of desire rather than another.” ([7] p. 91). Does this mean that Frankfurt demands too much reflection? Could it be that we would never be able to meet these requirements and that we would be completely taken over by our habits without having a real “say”? Do our habits turn us into wantons, i.e. in beings that are not self-directed, that do not care which desire moves them to act? This is only plausible under the assumption, that our habits are entirely unreflected. This may be because the self does not care about its own desires or because there is no such self at all. However, taking reflection seriously does not demand constant reflection of desires. What it demands is rather a coherence of desires that could be identified by reflection, when so engaged. Just as we don’t deny somebody to know X if he doesn’t constantly think of X, we cannot determine habits as outright unreflected. We don’t have to assume a single cognitive event on which we finally decide. Contrary to criticism, the theory rather requires reflection not to be overdone: Persons who constantly and comprehensively reflect upon their desires would risk of becoming incapable of any decision or action through excessive reflection in the sense of a compulsive disorder. After all, we must not pose a false dichotomy: in everyday life, there is also a considerable amount of actions which we cannot categorically call free or unfree. It makes no sense to say that looking on the ground while walking or shaking somebody’s hand are neither compelled (or instinctive) nor fully free—even though—and this is crucial for us as persons – we could start to reflect over doing such things if we would like to.

The fourth objection is a critique of a different type that has been recently expressed by Ahlin [46]. He criticizes Frankfurt’s approach on the grounds that it is “impractical” as it fails according to Ahlin to “reliably determine whether valid endorsement is actually taking place when the desire-holder is in that state of mind. To do so would require access to advanced (and currently unavailable) neuro-imaging technology, in addition to an in-depth knowledge of the psychological nature of endorsement” [46]. In our opinion, this objection can be easily rebutted: it does not make sense to conclude that a person X does not expresses a reflective authentic desire because this cannot be proven by brain activities. A sufficient indicator of authenticity would be to establish during a well led traditional physician-patient encounter that patient X adopts a reflective position. It would be quite absurd to assume that a patient lacked autonomy, because this could be read off the brain, even though a conversation by the tricks in the book would have turned out to be the opposite. This kind of “freedom” is reminiscent of Gilbert Ryle’s famous dictum of the “ghost in the machine” - except that in the case of the critique of Frankfurt’s model, the (allegedly) true free ego is sought in the brain. But there is no additional type of reflectiveness in the brain that a person would not be aware of and that can only be accessed via brain imaging or other types of technology showing brain activities.

Since all objections discussed above can be refuted, we believe Frankfurt’s theory to be compelling, and we will see in the following section how it helps us to understand various types of compulsion better, and how it can be integrated into the understanding of DMC.

### Inner and outer constraints and the patient’s appreciation

The importance of the absence of constraints for DMC has often been highlighted [12, 47, 48]. However, whereas these contributions have focused on external constraints, Frankfurt’s theory elucidates the relevance of inner constraints. At first, “inner” and “outer” are just metaphors. How could we make sense of it? Under normal circumstances, an outer constraint is an obstacle for freedom of action. It makes it impossible to act as we want like we have seen above in the prisoner-example. However, the outer circumstances could very easily impact the will. In the case of the prisoner, she could—after a long time in prison—resign or even give up thoughts about escaping the prison at all. This does not necessarily mean unfree will, but perhaps rather a reasonable adaption of the will to the present situation. We can draw an analogy to a severe physical illness: For example, a paraplegia considerably restricts a patient’s mobility. Knowing that, the patient may give up his prior wish to run a marathon. This change of mind may be perfectly free, although in the same time regretted very much. It demonstrates the patient’s appreciation of his situation and the ability to identify with his own will in view of the circumstances. But again, the causal history is quite obvious: without the paraplegia, the patient would have most likely kept up his wish. If, however, the patient was overwhelmed by pain, if he was deeply desperate and hopeless in a way that made it impossible for him even to pay attention to the remaining options, he would be unfree. It would be impossible for him to consider his own desires in a sufficiently open manner. In this case, the “outer” constraint would have led to an “inner” one which destroys free will.

It is evident that the line cannot be easily drawn between a sufficiently reflected adjustment to circumstances – which is free – and a retreat or “breakdown” of the will facing compulsory circumstances. Though, we should not expect the theory to solve all the problems in one fell swoop anyway, but to make it possible to describe and argue more accurately. We are familiar with this distinction when we think of living organ donation. In many cases, it is very difficult to judge for what reason someone is donating, even for the donor herself. We should not only concentrate on intended compulsion by any other person (what would be the case if we follow Beauchamp who determines compulsion as an intentional act of someone else [7], but also carefully assess the coherence of desires of the donor. It is decisive whether anybody can “identify with her motivating desires” or “if she is so alienated from them that she regards the conditions necessary for identification as a constraint on what she has reason to do.” ([49] p. 44). Therefore, freedom of the will, and consequently appreciation, is not so much a matter of influence by others, but identification with the relevant desire. It is this need to understand the full meaning of one’s own desire which makes free will an aspect of the appreciation criterion of DMC.

In summary, a will cannot be free when persons are compelled or manipulated in their wishes and opinions in a way that leads to a will that seems alien to her or him. For a wish to be non-alien, a person must be able to apply the information to his or her own situation, which precisely means to appreciate. This, of course, requires an ability to understand one’s own wishes and demands, so to speak—as well as those of third-parties—about what is at stake when deciding what to do, because *there is no appreciation without understanding*.

Attention to free will, according to the compatibilist understanding defended in this paper, therefore helps to fill gaps that might arise in the application of the classical criteria for DMC, because the embedding of a wish into the higher-order desires—the desires that express who we are and what we want to be—such as life plans or overall goals, are not sufficiently taken into account. In the last section, we turn to the question, how this “embedment” could be examined more closely in a clinical setting.

### A sensitive evaluation of DMC: incorporating the relation between desires

It is interesting to note that Frankfurt explains his theory in terms of pathology. Let us briefly look at his rich characterization of these phenomena. He describes the threat to our freedom as “psychic analogues of the seizures and spasmodic movements that occur at times in our bodies” and as “things that happen to us” [38]. As persons, we experience that “some of the psychic raw material that we confront may be so objectionable to us that we cannot permit it to determine our attitudes or our behavior.” ([38] p. 9). Freedom of the will then consists in opposing these unwelcomed thoughts: “we deny them [intruding thoughts] any entitlement to supply us with motives or with reasons.” ([38] p. 10) and we are defeated, when “the outlaw imposes itself upon us without authority, and against our will.” [38].

Consider the example of a patient with a pathological fear of general anesthesia as a specific phobia. This patient must undergo a specific surgical procedure involving general anesthesia in order to treat a certain disease. The patient decides to refuse the surgical procedure because of his or her strong fear of general anesthesia. The patient is completely aware of this strong fear being irrational and one of the diagnostic criteria for the specific phobia. Here, the action-governing first-order desire is to refuse the surgical procedure because of the strong fear of general anesthesia. However, the patient’s second-order desire is not to have the first-order desire because it is unreasonable. Therefore, the patient lacks free will because he does not succeed in rendering the first-order desire ineffective. The only way to clarify whether the rejection of any surgical procedure is a direct consequence of the phobia is to consider the system of desires, especially on the second-order level. On this level, conflicts could easily be overlooked, if attention is only paid to the obvious well-informed first-order desire, which is, in consideration of the fear, perfectly rational. It might be that the patient’s second-order desire consists in not wanting anesthesia at all. However, it is likely that the patient has another second-order desire to be in good health and live well which could be explored in a conversation about his core values. Therefore, if patients want to have the will to foster their health condition (and they know that and are able to express themselves), then, in order to exhibit free will, this desire should overrule other desires rising from the phobia that are certainly impeding the attainment of these goals. Unless the surgery is considered as unpromising or even pointless, these patients should be supported in taking into account the potential consequences of a treatment refusal and to give their informed consent accordingly.

Furthermore, the acceptance of Frankfurt’s approach as a guiding interpretation is convincing because it well captures approaches with different theoretical assumptions. In the following, we show this by means of two clinical case examples: Meynen introduces the (fictional) case of Ms. X, a patient suffering from obsessive-compulsive disorder (OCD) [23], who strictly cleans her house for several hours a day, while it would cause enormous distress for her not to do so. Frankfurt’s analysis fits very well: On the one hand, Ms. X wants things to go on as they are but still suffers under the OCD. She experiences competing first-order desires, and, as Meynen describes the case, she also has the wish to refrain from OCD which is not offhand recognizable and why a second-order desire is conceivable. Meynen portrays patients like Ms. X as feeling “completely alienated from their behavior, not considering it their behavior anymore” ([23] p. 327). The treatment should be about the recognition of these higher-order desires in light of what the patients fundamentally care about. Accordingly, DMC depends on the ability to recognize these specific desires.

In another intriguing contribution, Banner and Szmukler investigated the meaning of objectivity in evaluating DMC following ideas of Donald Davidson [50]. The authors deal with cases in which patients express wishes which do not fit with their earlier statements or which appear to be bizarre or unfounded to others. Banner and Szmukler therefore call for consideration of “the context in which such beliefs arise” and to see how they connect with “other beliefs, values and behaviours” ([50] p. 358). Their presented cases show that even very unusual, highly immoral, or repulsive wishes can be freely willed, provided that they fit coherently into the patient’s system of values and beliefs. These are, however, precisely these cases in which the patient is often denied DMC without good reason [50]. Such internal coherence of desires and traceability to more fundamental goals, values, or beliefs can be modeled very well within the Frankfurtian framework.

The core concern seems to be that in evaluating DMC, higher-order desires such as life plans, values, beliefs, or overall goals have to be felt out. However, the most important and likewise simple question is which desires patients want to have. Or in a wider sense, what kind of person would they like to be and whether a certain desire can be interpreted as an expression of the person’s self. As pointed out, this doesn’t demand complete self-knowledge (which is beyond human capacity). Thus, the evaluating physician shouldn’t only ask what patients want with regard to their actions (first-order desires) but also what someone wants to be his or her will. Doing this, physicians have to be sensitive to mental disorders that can impair the formation of wishes and wills, even though a disease does not necessarily exclude that a patient has a harmonious will, i.e. the second-order desire concords with first-order desires, and where he consents to a treatment. There has to be a relevant degree of psychopathology to cause a truly unharmonious will that goes beyond “normal” functioning.

## Conclusions

Even though free will is not an additional criterion for DMC, the common understanding of the appreciation criterion shows gaps because certain types of lacking free decision-making that are the consequence of a non-harmonious will are not addressed clearly enough. We have drawn attention to the importance of making patients aware of and have them think about the question as to whether their first-order will is something they also wish on the second-order level because that is precisely what is needed to determine whether their will is free. Following Frankfurt [38–41, 44], the insight is that patients not only have wishes related to actions or concrete types of behavior, but also wishes that concern other wishes some of which express essential qualities of the person herself. If the physician who evaluates a patient’s DMC does not identify broad and unsolvable conflicts between the patient’s wishes related to actions *and* those related to wishes, he can conclude on good grounds that the patient’s will is free (harmonious), independently of what the content of the will is.

This does not lead to additional difficulties or a higher threshold for DMC and thus informed consent but is important and useful to analyze more appropriately the processes that lead to the forming of a will and to examine whether and why in acute situations patients may have difficulties to form and express an authentic, i.e. harmonious, will. With the at hand depicted compatibilist model, a sensitive evaluation of DMC is realizable in clinical practice.

## Abbreviations

DMC: decision-making capacity
OCD: obsessive-compulsive disorder.
